# Hydrogen Diffusion in Deformed Austenitic TRIP Steel—A Study of Mathematical Prediction and Experimental Validation

**DOI:** 10.3390/ma17246114

**Published:** 2024-12-13

**Authors:** Christian Hempel, Marcel Mandel, Caroline Quitzke, Marco Wendler, Thilo Kreschel, Olena Volkova, Lutz Krüger

**Affiliations:** 1 Sunfire GmbH, Gasanstaltstr. 2, 01237 Dresden, Germany; christian.hempel@sunfire.de; 2 Institute of Materials Engineering, Technische Universität Bergakademie Freiberg, Gustav-Zeuner Str. 5, 09599 Freiberg, Germany; krueger@ww.tu-freiberg.de; 3 Institute of Iron and Steel Technology, Technische Universität Bergakademie Freiberg, Leipziger Str. 34, 09599 Freiberg, Germany; caroline.quitzke@iest.tu-freiberg.de (C.Q.); marco.wendler@iest.tu-freiberg.de (M.W.); thilo.kreschel@iest.tu-freiberg.de (T.K.); olena.volkova@iest.tu-freiberg.de (O.V.)

**Keywords:** cold rolling, high alloy austenitic steel, hydrogen diffusion modelling, hydrogen embrittlement, TRIP

## Abstract

This study focuses on the effect of pre-deformation on hydrogen diffusion and hydrogen embrittlement of the high alloy austenitic TRIP steel X3CrMnNiMo17-8-4. Different cold-rolled steel sheets with thicknesses of ≤400 µm were electrochemically charged on both sides in 0.1 M sodium hydroxide with hydrogen for two weeks. Comparative measurements on uncharged and immersed samples prove that hydrogen causes embrittlement in this steel for all investigated states. The embrittlement increases with increasing pre-deformation and is accompanied by deformation-induced martensite formation. The corresponding fractured surfaces were examined using electron microscopy and compared to modelled hydrogen distributions with previously determined diffusion coefficients. For this purpose, various diffusion coefficients are determined using the Devanathan–Stachurski permeation test and hot extraction in order to describe the diffusion process. The hydrogen concentration profiles and the fractographic analyses show a good agreement, so this study provides a basis for estimating the embrittlement behaviour for later application.

## 1. Introduction

Hydrogen is said to be one of the key options for decarbonisation [1,2] to achieve the ambitious goals of the energy transition. In this context, new components and devices are being developed for hydrogen applications [3,4], but it is also conceivable to use existing systems [5]. Due to the fact that hydrogen is the smallest atom, it can penetrate into several materials, e.g., polymers [6], ceramics [7] or metals [8], and can also stimulate chemical reactions and subsequently generate a wide variety of corrosion products [9]. One of the pivotal questions is if and how dissolved hydrogen affects the used materials [10,11]. It is well known that hydrogen interacts with materials, especially metals, and can change the mechanical properties [12,13]. This effect, also known as hydrogen embrittlement (HE), is based on the hydrogen uptake and its diffusion behaviour, which depends on a large number of influencing factors [14,15,16]. As known for steels, susceptibility to HE is related to the apparent hydrogen diffusion coefficient. Ferritic steels that exhibit a relatively high diffusion coefficient (*D_app_*~2 × 10^−7^ cm^2^ s^−1^ [17]) are more susceptible to HE than austenitic steels (*D_app_*~3 × 10^−12^ cm^2^ s^−1^ [18]), which exhibit a significant lower diffusion coefficient [19]. For two- or multiphase systems, the diffusion coefficients are usually between those of the single components and depend on the phase composition [20,21].

With regard to modern steel development, weight and cost savings are often achieved through the use of advanced high-strength steels (AHSSs). This class of steels includes transformation-induced plasticity (TRIP) steels that show a deformation-induced martensite formation from metastable austenite during plastic deformation [22]. This phase transformation leads to enhanced mechanical properties, e.g., higher strength [23,24]. According to this phase transformation, TRIP steels appear as two- or multiphase systems. The use of such steels in hydrogen applications is attractive because of their good mechanical properties, such as high strain hardening, high yield and tensile strength and fracture toughness [25]. Investigations on the HE of pre-strained TRIP steels have already been part of the literature [26,27,28]. In general, all studies agree that a pre-deformation leads to a higher susceptibility to hydrogen embrittlement.

In addition to classic material testing, theoretical calculations for describing HE have also been established [29,30,31,32]. With such calculations, the fracture behaviour can be characterised, and an estimation of the durability of materials under real conditions is possible, which is a main task when making material decisions.

An essential part of this study is to investigate the effect of cold working/phase transformation on susceptibility to HE of the newly developed TRIP steel X3CrMnNiMoN17-8-4. The steel is characterised by excellent strength, formability and corrosion resistance, which makes it very interesting as a pipeline material for applications in the oil and gas industry. For this purpose, different pre-strained material states were electrochemically charged with hydrogen and subsequently analysed by tensile tests. In addition, extensive experiments were carried out using hot extraction in order to describe the hydrogen distribution after charging and, finally, at the stage of mechanical testing. The results of hot extraction were compared to calculations of the hydrogen distribution along the steel cross section and finally correlated to fractographic analyses of the fracture surfaces.

## 2. Materials and Methods

### 2.1. Materials

In this study, the high alloy austenitic steel X3CrMnNiMoN17-8-4 was investigated, and its chemical composition is given in Table 1. A precise description of the manufacturing process is depicted in detail by Quitzke et al. [33].

In order to achieve thin steel sheets, a hot-rolled strip of thickness *h* = 4 mm was cold-rolled in a quarto stand. Due to work hardening, intermediate stress relief annealing was necessary after several rolling passes. However, the actual starting points after the last solution annealing are summarised in Table 2. The given true strains *ε* were calculated by Equation (1) as follows:

(1) $$\varepsilon =\left|\ln\;\frac{h_{1}}{h_{0}}\right|$$

where *h*_0_—thickness before rolling; and *h*_1_—thickness after rolling.

### 2.2. Hydrogen Charging

Samples with a dimension of 55 × 55 mm were electrochemically charged with hydrogen for two weeks in a double-cell test set-up, according to Devanathan and Stachurski (DS cell) [34]. Before charging, all samples were coated with palladium to prevent oxide layers and anodic metal dissolution [35,36]. The permeation experiments were carried out in a 0.1 M sodium hydroxide solution within both cells. In order to reduce hydrogen recombination, an amount of 25 mg L^−1^ of arsenic oxide was added as recombination poison [37]. The charged area was *A* = 13.46 cm^2^ (*d* = 4.14 cm). A current density of *i_c_* = −10 mA cm^−2^ was applied on both sides to achieve a uniform hydrogen distribution within the steel samples.

Furthermore, various samples with a pre-deformation of *ε* = 0.49 were charged for 4, 7, 14, 21 and 36 days. The total amount of diffusible hydrogen was determined using hot extraction (see Section 2.5) and compared to the mathematical calculations. In addition to the determination of hydrogen concentration during charging, the hydrogen amount for the 36-day charged sample was also determined during discharging after 3, 7, 14, 28 and 56 days. Therefore, the charged sample was cut into pieces, and the extraction was performed as triple repeat determination. Similar tests were performed for the other material states.

### 2.3. Tensile Tests

**Figure 1:**
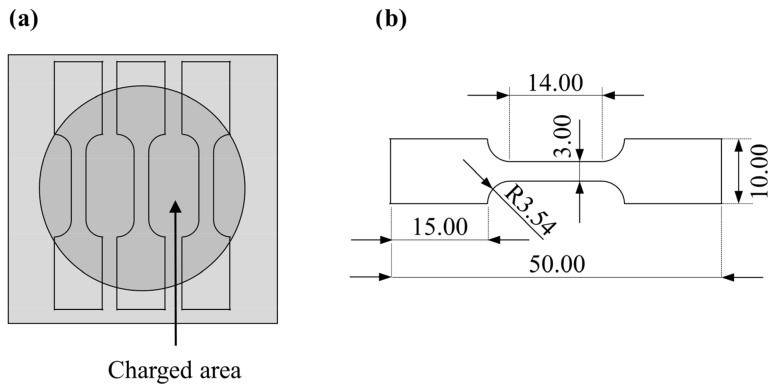
(**a**) Schematic drawing of tensile test samples extracted from the hydrogen-charged area; (**b**) geometry of the used tensile samples according to [38] (Unit:  mm).

Three samples were cut from the charged area using electrical discharge machining, as schematically shown in Figure 1a. The sample geometry is given in Figure 1b. The tensile tests were performed using a miniature load frame (Kammrath & Weiss, Schwerte, Germany) at a linear velocity of 10 µm s^−1^. The corresponding strain measurements were evaluated by an optical extensometer based on digital image correlation using the microDAC system (Chemnitzer Werkstoffmechanik, Chemnitz, Germany). Three different experimental conditions were chosen to examine the influence of hydrogen charging as well as the influence of corrosion caused by the electrolyte. The first one was the uncharged initial state, the second one was the two-week hydrogen-charged state and the third one was tested after two weeks of immersion in the 0.1 M sodium hydroxide solution. Due to the necessary manufacturing process, the mechanical tests were carried out two weeks after the charging was stopped so that the effusion of hydrogen was considered as well. Based on the high hydrogen charging test times at room temperature, the condition of a two-week hydrogen charging was chosen as an average testing time, which should show a dependence of the embrittlement depth on the degree of deformation. The fractographic analyses carried out after the tensile test reveal this dependence very well. It is assumed that if the charging time is extended, the embrittlement front penetrates deeper into the material, the entire sample cross section becomes homogeneously brittle and it becomes more difficult to demonstrate the dependence on the degree of deformation. Nevertheless, varying environmental conditions and the extension of the charging time are recommended to gain a deeper understanding of the influence of hydrogen on TRIP steels. However, these far-reaching analyses are not the subject of this study.

### 2.4. Fractography

For the fractographic SEM analyses, a MIRA 3 XMU (TESCAN, Brno, Czech Republic) was used to analyse the HE and to compare the size of the embrittled areas using mathematical calculations.

### 2.5. Hydrogen Determination

The amount of diffusible hydrogen was determined via hot extraction using nitrogen as carrier gas at 1373 K in a Rosemount H2A (Fisher-Rosemount, Langenfeld, Germany) gas analyser. For quantification, a thermal conductivity detector was used.

## 3. Mathematics

Based on the experimental investigations and results, a calculation of the hydrogen concentration profiles in dependence on the degree of deformation was performed. The nomenclature of the used parameters is given in Table 3.

The basis for the calculations of the diffusion processes are Fick’s laws I and II. The relation between sheet thickness (*L* ≤ 400 µm) and the hydrogen-charged area with a diameter of *d* = 4.2 cm allows for an assumption of a unidirectional diffusion along the sheet cross section. The location-dependent Fick’s law I in one dimension is given in Equation (2), and the corresponding Fick’s law II, which describes the change in concentration with respect to the time, is given in Equation (3).

(2) $$j\left(x,t\right)=-D\;\frac{\partial c(x,t)}{\partial x}$$

(3) $$\frac{\partial c}{\partial t}=-D\;\frac{\partial^{2}c(x,t)}{\partial x^{2}}$$

For a both-sided charging of plane sheets, an analytical solution based on a Laplace transformation that considers particular boundary conditions was stated by Crank [39] and is given in Equation (4). The boundary conditions are defined by the following:

1. A uniform hydrogen distribution in the material at the beginning of charging.
2. A symmetric and steady concentration of hydrogen at the entry sides.

(4) $$\frac{c\left(x\right)-c_{0}}{c_{1}-c_{0}}=1-\frac{4}{\pi }\sum_{n=0}^{\infty }\frac{{\left(-1\right)}^{n}}{2n+1}exp\left(-\frac{D{\;\left(2n+1\right)}^{2}\pi^{2}t}{4L^{2}}\right)cos\left(\frac{\left(2n+1\right)\pi x}{2L}\right)$$

where −L<x<L, and for a given diffusion coefficient and sample geometry, a concentration profile can be calculated for a specific charging time.

As mentioned above, the tensile tests could not have been performed during or immediately after charging. Therefore, it is also necessary to evaluate the discharging process of hydrogen. For this purpose, Equation (5), which is also given by Crank [39], was applied to describe the changes in concentration for a given initial hydrogen distribution. The equation is valid if the hydrogen concentration at the entry/exit side is zero, which is assumed when the charging process is stopped.

(5) $$c^{’}\left(x\right)=\frac{2}{L}\sum_{n=1}^{\infty }sin\left(\frac{n\pi x}{\pi }\right)exp\left(-\frac{D\pi^{2}n^{2}t}{L^{2}}\right)\int_{0}^{L}c\left(x\right)sin\left(\frac{n\pi x}{\pi }\right)dx$$

**Figure 2:**
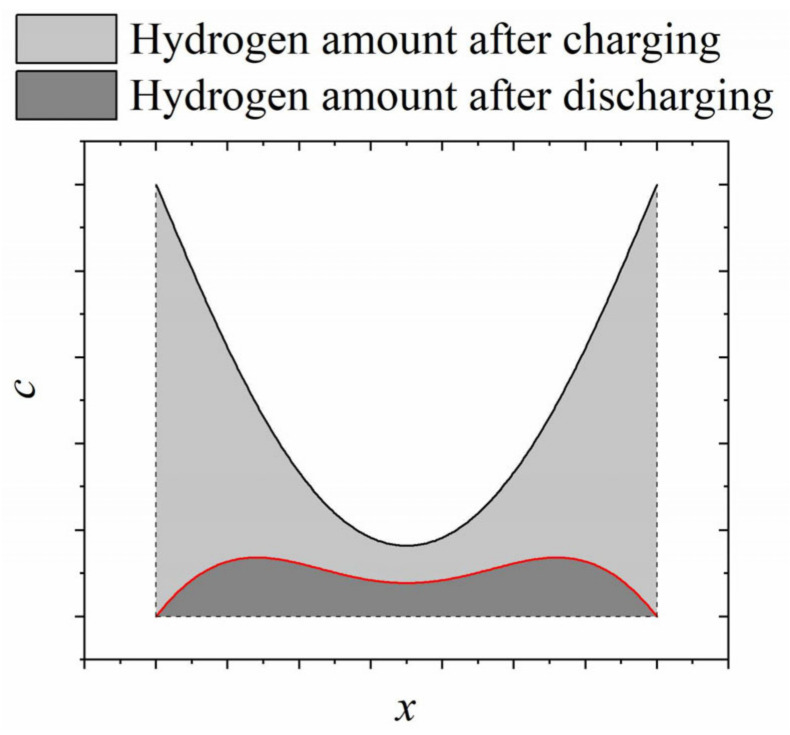
Schematic view of calculated hydrogen distributions in a plane sheet after hydrogen charging and subsequent discharging, grey areas indicate the integral of curves, representing the total amount of hydrogen. Black/red line—concentration profile *c*(x) after charging/discharging.

Finally, when using the calculated concentration profiles, the total amount of dissolved hydrogen is evaluable by integration (Equation (6)). In this study, the integrals were approximated via the trapezoidal method. Figure 2 shows an example of the calculated hydrogen distribution in a plane sheet after hydrogen charging and subsequent discharging. In this study, the step size of the position vector was chosen to be 1 µm for each calculation.

(6) $$c_{H}=\int_{0}^{L}c\left(x\right)\;dx$$

All calculations were determined using the MATLAB software R2018b by MathWorks (Natick, MA, USA). In conjunction with sum expressions, the Symbolic Math Toolbox was used as an additional tool. Our own designed MatLab scripts were used for the optimisation calculations based on the algorithm of Lagarias et al. [40]. Moreover, the calculations were carried out at room temperature and required the apparent diffusion coefficient *D_app_* and the hydrogen equilibrium concentration *c*_1_ as input parameters. To evaluate *D_app_*, hydrogen permeation tests at increased temperatures of 323 K, 338 K and 353 K were performed using a Devanathan–Stachursky test set-up, and the apparent diffusion coefficient at room temperature was extrapolated using the Arrhenius relationship. Details of the method are described elsewhere, and the interested reader is referred to ref. [41]. In a similar manner, *c*_1_ was extrapolated, applying Equations (4) and (6) to experimental results for the non-equilibrium state.

## 4. Results and Discussion

### 4.1. Modelling of Hydrogen Distribution and Determination of Hydrogen Concentration

The parameters necessary for modelling the hydrogen distribution in the samples are partially published elsewhere [41]. The pre-exponential factor and the activation energies were determined in a DS cell experiment for a temperature range from 323.15 K to 353.15 K in order to shorten the test duration. The Arrhenius equation (Equation (7)) is afterwards used to extrapolate the diffusion coefficient at room temperature (RT = 293.15 K). The calculated values are summarised in Table 4.

(7) $$D_{app}=k_{0}\cdot exp\left(-\frac{E_{a}}{R\text{·}293.15\;K}\right)$$

The initial hydrogen concentration was determined to be *c*_0_ = 0.93 ± 0.27 ppm and assumed to be uniform. The value was subtracted from the data, which were determined by hot extraction measurements.

**Figure 3:**
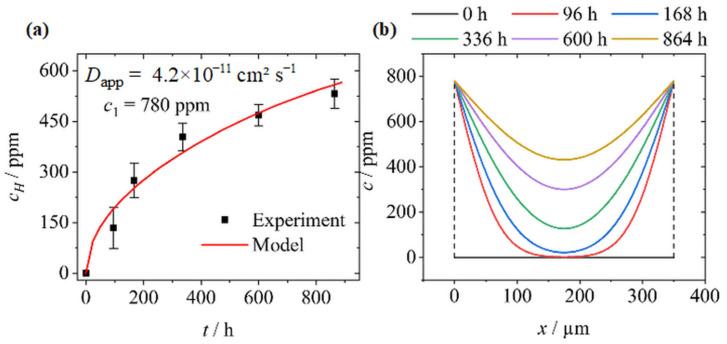
(**a**) Hydrogen concentrations in *ε* = 0.49 pre-deformed X3CrMnNiMoN17-8-4 and corresponding mathematical fit using Equations (4) and (6); (**b**) calculated hydrogen distribution profiles in pre-deformed *ε* = 0.49 samples after different durations of charging at RT using the extrapolated equilibrium concentration *c*_1_ = 780 ppm and the extrapolated apparent diffusion coefficient *D_app_* = 4.2 × 10^−11^ cm^2^ s^−1^.

In order to verify the extrapolated diffusion coefficients, various samples of the pre-deformed material state *ε* = 0.49 were charged with hydrogen for different periods, and the total amount of dissolved hydrogen was measured. Figure 3a shows the experimental data of these measurements. Due to the fact that the equilibrium was not reached, the value of the equilibrium hydrogen concentration *c*_1_ was fitted to the experimental data by substituting Equation (4) in Equation (6) and by using the extrapolated apparent diffusion coefficient at RT. The equilibrium hydrogen concentration was determined to be about *c*_1_ = 780 ppm. With respect to the fact that hydrogen concentrations higher than 1000 ppm have been reported for pure austenitic steels under similar charging conditions [42], the given value is reasonable. Figure 3b represents the corresponding hydrogen distributions that were calculated by Equation (4) for the different charging times in consideration of *D_app_* and *c*_1_.

**Figure 4:**
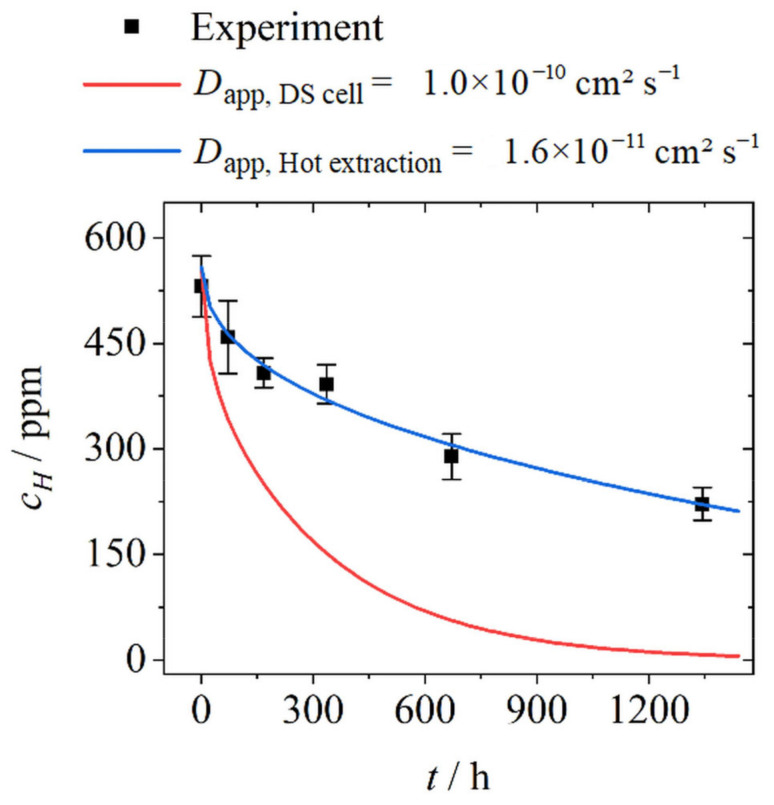
Experimentally determined hydrogen concentrations during discharging at atmosphere in *ε* = 0.49 pre-deformed steel sample after 36 days of hydrogen charging and corresponding mathematical fit using the extrapolated apparent diffusion coefficients *D_app_* determined by DS cell (red line) and by hot extraction (blue line).

Figure 4 shows the measured hydrogen amount during discharging in samples that were charged for 36 days and stored in a desiccator. As described in Section 3, Equations (5) and (6) were used to calculate the theoretical hydrogen distributions and overall hydrogen concentrations during the effusion of hydrogen. In the same way, as for the charging process, the Arrhenius equation (Equation (7)) was used to extrapolate the apparent diffusion coefficient at RT for the effusion of hydrogen. The calculated values from the DS cell experiments are summarised in Table 4. When comparing the values of hydrogen, determined via hot extraction (Figure 4, black squares), to the calculated outward diffusion using *D_app_ =* 1.0 × 10^−10^ cm^2^ s^−1^ of DS cell experiments (Figure 4, red line), it is noticeable that there is a clear deviation. When using the DS cell, the measured hydrogen effusion was much faster than during storage at low humidity atmosphere. There are two possible explanations for this discrepancy. On one hand, mainly weakly bound hydrogen is detected in the DS cell measurements, e.g., hydrogen that is interstitially dissolved in the lattice. Whether hydrogen diffuses out of traps during discharging in the DS cell, and if so, how much hydrogen, can hardly be said without further methods. Due to the higher binding energy, trapped hydrogen diffuses more slowly out of the samples. On the other hand, the both-sided charged samples that were used for hot extraction were cleaned after their removal and stored in low-humidity air. Larger parts of the palladium layer were detached during cleaning, whereby it can be expected that a passive oxide has subsequently formed on the underlying metal surface. The literature provides diffusion coefficients for oxides that are some orders of magnitude lower even than that for austenite, e.g., $D_{Cr_{2}O_{3},RT}\approx \;8\;\times 10^{-18}\;{\mathrm{cm}}^{2}{\;s}^{-1}$ [43]. Fe_2_O_3_ is described as a nearly entire diffusion barrier for hydrogen [44]. In order to obtain an impression of the hydrogen effusion actually taking place, Equations (5) and (6) were fitted to the experimental data by variation of *D_app_*, resulting in a diffusion coefficient of *D_app, Hot extraction_* = $1.6\times 10^{-11}\;{\mathrm{cm}}^{2}{\;s}^{-1}$ that describes the experimental findings much better (Figure 4, blue line).

Using the results of the hot extraction experiments, the apparent diffusion coefficients for the states *ε* = 0, 0.32 and 0.39 were determined in the same way. The values are summarised in Table 5.

**Figure 5:**
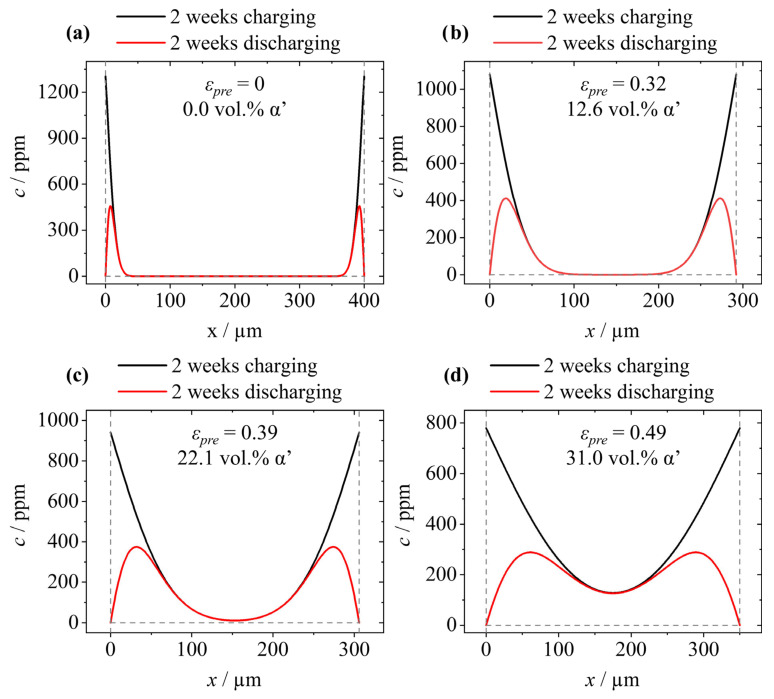
Hydrogen distributions in different pre-deformed states of X3CrMnNiMoN17-8-4 after two weeks of electrochemical charging (black line) and after two weeks of discharging (red line); (**a**) *ε* = 0, (**b**) *ε* = 0.32, (**c**) *ε* = 0.39, and (**d**) *ε* = 0.49. Dashed lines indicate the sample width used for calculation.

For the calculation of the hydrogen distribution profiles after two weeks of charging, the diffusion coefficients provided in Table 4 and Equation (4) were used. The values provided in Table 4 were determined in the preliminary study by Ref. [41] and exhibit a significant drop for the pre-exponential factor *k*_0_ for *ε* = 0.49. It is assumed that this effect is due to the increased martensite content and, consequently, higher electrochemical corrosion reaction and passive oxide formation, which ultimately hinders the hydrogen diffusion process. The diffusion coefficients given in Table 5 and Equation (5) were used to calculate the concentration profiles after two weeks of discharging, which corresponds to the time of the tensile tests. The results are shown in Figure 5. The hydrogen distribution profiles prove that the penetration depth increases when the degree of deformation increases. This effect is related to the increased diffusion coefficient of hydrogen in martensite [45] when compared to austenite [42]. The profiles also confirm that the hydrogen remained in the samples even after the two weeks of effusion.

Based on the distribution profiles, the calculated and experimental total amounts of hydrogen *c_H,calc_* and *c_H,exp_* after two weeks of charging and two weeks of discharging were determined by Equation (6). The results are summarised in Table 6. The comparison shows good agreement between calculation and experiment and confirms the appropriateness of the chosen mathematical approach.

### 4.2. Mechanical Testing

**Figure 6:**
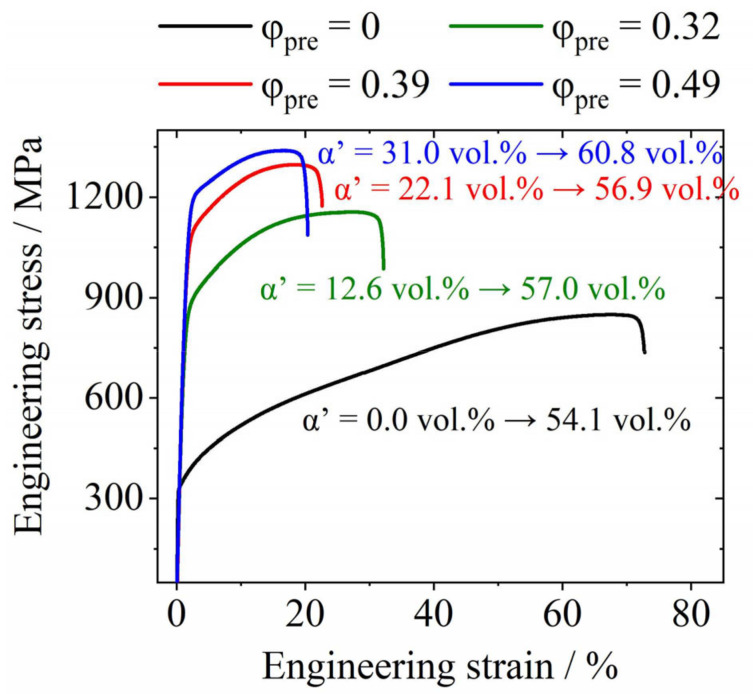
Results of tensile tests for X3CrMnNiMoN17-8-4 at different states of pre-deformation. The values of *α*′ provided for each curve give the initial α′ content received after cold rolling and the final *α*′ content determined after tensile testing.

The engineering stress–strain plots of the uncharged initial state and pre-deformed material states are presented in Figure 6, indicating a significant change in mechanical properties due to the pre-deformation. The pre-strain effectively enhances both yield strength (YS) and ultimate tensile strength (UTS), whereas the ductility decreases, reflected in a decrease in total elongation (TE).

During pre-strain, the dislocation density increases, and parts of the metastable austenite transform to α′-martensite [33,41,46,47,48,49]. Both dislocations and the new interfaces interfere with dislocation movement and cause an increase in YS and UTS. The strain-induced martensite has much higher strength than austenite and less ductility. The decrease in ductility and, thus, in TE also results from impeded dislocation movement possibilities. In addition, it is noticeable that the total ratio of α′-martensite after pre-deformation and tensile tests increases with higher degrees of previous cold rolling. The observation is explained by the fact that cold rolling does not represent a uniaxial load. Thus, the values of true strain until the samples fracture and the amount of α′-martensite slightly increase.

**Figure 7:**
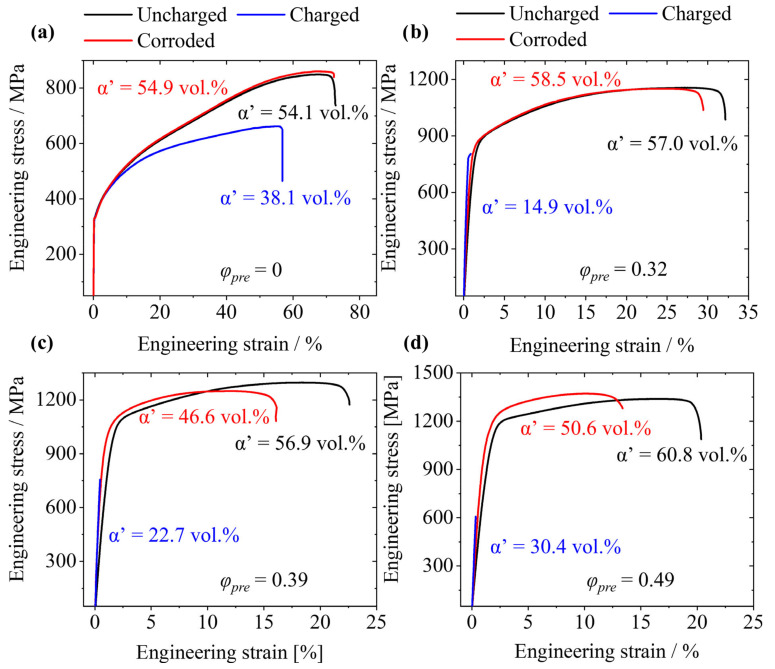
Tensile test results for X3CrMnNiMoN17-8-4 with and without hydrogen charging and after 2 weeks of immersion in 0.1 M sodium hydroxide solution with different pre-deformation; (**a**) *ε_pre_* = 0, (**b**) *ε_pre_* = 0.32, (**c**) *ε_pre_* = 0.39, and (**d**) *ε_pre_* = 0.49.

Figure 7 presents the engineering stress–strain curves of the samples that were uncharged, hydrogen charged in 0.1 M sodium hydroxide for two weeks and immersed in 0.1 M sodium hydroxide for two weeks. For the undeformed state *ε* = 0 (see Figure 7a), it is clear that immersion in 0.1 M sodium hydroxide solution does not have a significant effect on the mechanical properties. The result confirms the good corrosion resistance of CrNi steels, which is also proved by electrochemical tests in the literature [50]. When comparing the annealed state to the cold-worked states, an opposite effect is noticed. In particular, TE decreases as a result of immersion of the pre-deformed samples in the sodium hydroxide solution (see Figure 7b–d). It is known that the corrosion resistance decreases as a result of plastic deformation [51,52]. A similar observation is made in this study, as TE decreases stronger in dependence on pre-deformation in comparison to the not immersed state and also increased pre-deformation. The α′-martensite that is induced during cold rolling promotes a pile-up of dislocations. This causes higher local potential differences that are associated with higher corrosion susceptibility. Kolodii [53] also confirms this observation by thermomechanical calculations. The corrosive attack leads to microcracks, which in turn leads to localised stresses that finally promote a failure during the tensile test. In addition, the corrosive attack also promotes the formation of hydrogen, which can embrittle the material as well. The corrosive attack also reveals that the applied palladium coating cannot be considered as perfectly dense. Nevertheless, it should also be noted that the effect of corrosion is noticed in the range of higher elongations. YS and UTS do not deteriorate significantly for the pre-deformed samples during two weeks of immersion.

In contrast, the hydrogen has a significant embrittlement effect on all material states (see Figure 7a–d). For the undeformed state, this is primarily reflected in a reduction in UTS and TE (Figure 7a). As shown in Figure 5a, the theoretically calculated hydrogen penetration reaches a depth of approximately 40 µm. Thus, hydrogen does not affect the entire cross section, and a ductile deformation occurs. The distinct increase in α′-martensite during tensile testing also shows that the samples are ductile even after being charged with hydrogen. When the pre-deformation further increases, the ductility decreases rapidly. For the material state *ε* = 0.32, the yield point is just reached, and there is even a slight increase in the martensite fraction. The two material states with the highest degree of deformation already break in the range of elastic deformation. The rupture stress even decreases for the pre-deformed states, though the tensile strength originally increased. It is stated that the embrittlement caused by hydrogen increases when the degree of deformation increases. Similar results were also obtained by Hojo et al. [26], who examined a pre-strained TRIP-assisted bainitic ferrite steel. Two main reasons are given for the obtained observations. The first reason is that the phase transformation from austenite (fcc lattice) to α′-martensite (bcc lattice) leads to a significantly higher apparent diffusion coefficient. Considering that hydrogen occupies tetrahedral voids in bcc [54] and octahedral in fcc [55], the faster hydrogen diffusion is a consequence. The occupied voids in the bcc lattice are smaller (lower binding energy [56]) and have a shorter distance to each other (lower migration energy [57]). As a result of a higher diffusion coefficient, hydrogen can penetrate the material more quickly and is able to reach more traps, which are primarily responsible for HE [58,59]. This, in turn, leads to a higher hydrogen-affected cross section affected and consequently to a higher susceptibility for HE. The second reason is that the pre-strain causes a significant increase in dislocation density, which acts as a hydrogen trap [60]. As Novak et al. [61] showed in a micro-mechanical model, dislocations can act as a main initiation point for an intergranular fracture during the mechanical loading of hydrogen-charged samples. It is concluded that after charging a material of high dislocation density, the number of hydrogen-filled dislocations increases, which finally increases the susceptibility to HE.

### 4.3. Fractographic Analysis and Comparison to Calculated Hydrogen Distribution Profiles

**Figure 8:**
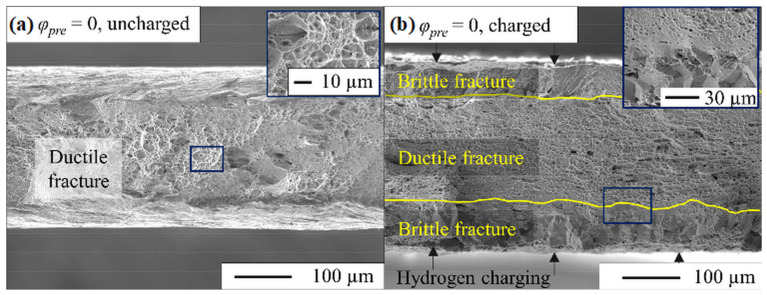
Comparison of fractured surfaces after tensile testing of the X3CrMnNiMoN17-8-4 in the (**a**) uncharged state and (**b**) after two weeks of hydrogen charging.

Figure 8a shows the fracture surface of the uncharged initial state. A ductile fracture is observed for all uncharged samples, which is expressed by the formation of dimples [62]. In contrast, Figure 8b shows the fracture surface of the undeformed state (*ε* = 0) after two weeks of hydrogen charging, revealing a ductile fracture in the centre of the sample and a brittle fracture at the top and the bottom where the hydrogen penetrated.

**Figure 9:**
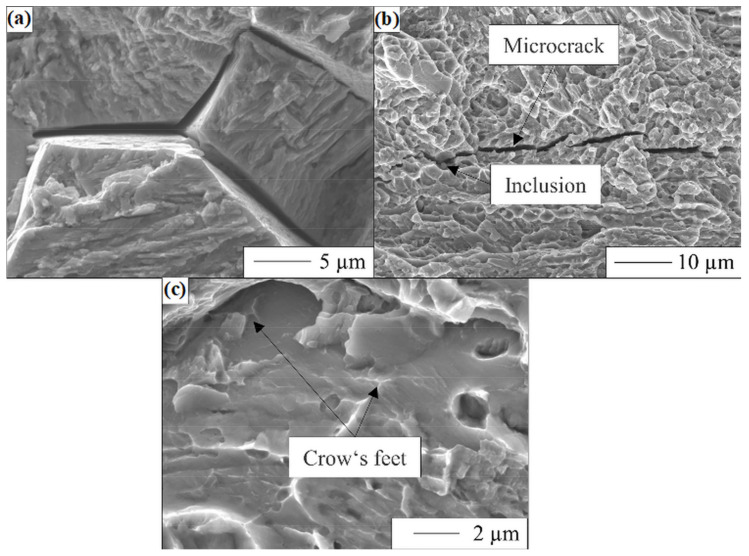
Characteristics of HE on the fracture surface of hydrogen charged X3CrMnNiMoN17-8-4 after tensile testing. (**a**) Gaping grain boundaries, (**b**) microcracks around inclusions, and (**c**) fine ductile hairlines known as “crow’s feet”.

The areas of brittle fracture show typical indications of hydrogen-assisted cracking, and in Figure 9, an overview of the main differences in ductile fracture is shown in detail. A significant characteristic is the gaping grain boundaries (Figure 9a), which are associated with intergranular fracture along prior austenite grain boundaries. This phenomenon is linked to the theory of hydrogen-enhanced decohesion (hereinafter HEDE) [63,64]. The HEDE assumes that hydrogen reduces the interatomic bonding force in metals between adjacent crystallographic planes. During the fracture propagation, hydrogen diffuses preferentially to energetically favourable areas (e.g., grain or phase boundaries), lowering the cohesive strength and surface energy. A further characteristic, also explained by the HEDE theory, is the microcrack formed along a non-metallic inclusion, shown in Figure 9b. Furthermore, residual ductility is observed on the grain surfaces and is known as “crow’s feet”, see Figure 9c, which is also typical for HE [65,66]. These fine plastic parts are explained by a local drop in yield stress due to hydrogen and an increased mobility of dislocation movement. The mechanism is known as hydrogen-enhanced local plasticity (HELP) [67,68].

**Figure 10:**
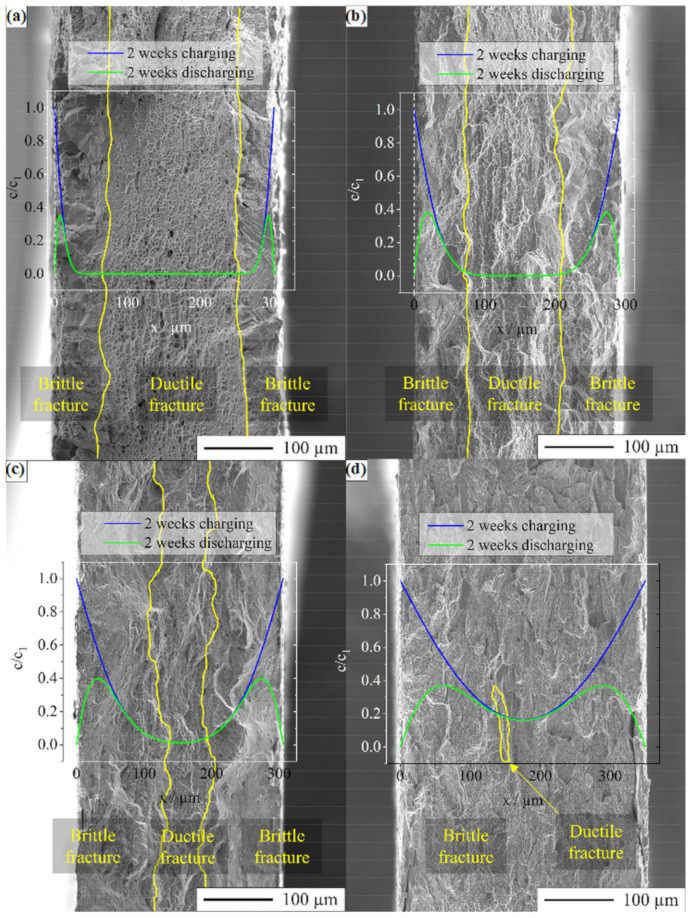
Comparison of fracture surfaces and calculated hydrogen distributions after two weeks of charging and two weeks of discharging in the samples of X3CrMnNiMoN17-8-4 at different degrees of deformation with (**a**) *ε* = 0, (**b**) *ε* = 0.32. (**c**) *ε* = 0.39, and (**d**) *ε* = 0.49.

Figure 10 shows the cross sections of the fractured surfaces of undeformed and pre-deformation states after two weeks of hydrogen charging. It is clear that the ratio of the brittle fracture surface compared to the ductile fracture surface increases when the degree of deformation increases. While many dimples are found in the undeformed state (Figure 10a), brittle fracture occurs almost over the entire cross section at the highest pre-deformed state with *ε* = 0.49 (Figure 10d). In principle, it is assumed that hydrogen propagates faster in the deformation-induced martensite due to its higher diffusion rate.

These observations are in good agreement with the calculated concentration profiles presented in Figure 5. Overlaying the profiles with the fractographic analyses exhibits the best correlations for the pre-deformed states (Figure 10b–d), as these show little or no plastic deformation. The discrepancy of the undeformed state (Figure 10 a) is related to the determination of the diffusion coefficient on a significantly thinner metal sheet (see Ref. [41] with *h*_0_ = 80 µm) that was used for the concentration profile calculation. Although the chemical composition is almost identical, differences in the grain size distribution of the sample used in Ref. [41] and the sample used in this study influence the hydrogen diffusion behaviour significantly. In addition, the charged samples of the initial state also show plastic deformation and as a result, the thickness of the cross section changed. The penetration depth of the hydrogen was simply assumed to be constant in Figure 10a, but it does not correspond to reality. For this reason, the hydrogen concentration profiles of the undeformed state are considered a rough approximation.

It is important to mention that the fractographic images exhibit a very homogeneous hydrogen embrittlement front and penetration depth on both sides for all degrees of deformation. This characteristic is clearly attributed to the experimental procedure used for the hydrogen charging in the DS cell. The homogeneity of the electric field, as well as the high current applied during the charging, promote a strong vertical hydrogen diffusion and show homogeneity in the material degradation behaviour, which afterwards can be theoretically calculated very well with the suggested approach.

Nevertheless, when transferring the results to the application, it is recommended to consider an inhomogeneity of the hydrogen distribution on the material surface and thus a local risk of embrittlement. Furthermore, homogeneous material deformation cannot be assumed under application conditions, so structure-related different penetration depths and thus local embrittlement effects can be more pronounced and must also be considered. As a consequence, the calculation of the concentration profile becomes significantly more demanding.

The consideration of inhomogeneous hydrogen distribution and a varying degree of deformation within the material are the subject of future research initiatives, whereby the finite element method (FEM) is also used as a mathematical tool for theoretical calculations.

## 5. Conclusions

In this study, the effect of both-sided electrochemical hydrogen charging on the mechanical properties under the influence of cold working on TRIP steel sheets of thicknesses ≤ 400 µm was investigated. In addition, calculations based on DS cell and hot extraction experiments were carried out and compared to the results of fractography. The results of the study are summarised as follows:

- The susceptibility to HE increases with increasing pre-strain for the investigated steel sheets. The effect is attributed to the increased diffusion coefficient in α′-martensite and the increased dislocation density.
- DS cell experiments are useful for the description of hydrogen charging but not useful when calculating the real hydrogen effusion behaviour.
- Hot extraction experiments are adequate to calculate diffusion coefficients for hydrogen diffusion during charging and for the period between charging und mechanical testing.
- The determined diffusion coefficients are used to calculate the hydrogen distribution profiles at the moment of tensile loading. The depth of penetration in the concentration profiles corresponds well to the brittle fracture in the fractographic sample analysis.
- The lower the pre-deformation of the steel and thus the deformation-induced martensite, the lower the hydrogen penetration depth.

## Figures and Tables

**Table 1:** Chemical composition of the investigated high alloy austenitic stainless steel in wt.%.

C	Cr	Mn	Ni	Mo	Si	N	Fe	Traces
0.03	17.4	7.48	3.63	0.56	0.15	0.19	bal.	S, P, Al, Nb, Ti, Sn < 0.01 Co, Cu, W < 0.03 V < 0.06

**Table 2:** Strip thickness before and after cold rolling with a corresponding number of roll passes and calculated true strain.

Strip Thickness *h*_0_ Before Cold Rolling [µm]	Strip Thickness *h*_1_ After Cold Rolling [µm]	Number of Roll Passes	True Strain
400	400	-	0
400	292	1	0.32
450	305	2	0.39
570	350	3	0.49

**Table 3:** Applied nomenclature in this study.

Symbol	Meaning
*A*	Charged area, 13.46 cm^2^
*c*	Concentration of hydrogen, wt.%
*c_0_*	Hydrogen concentration before charging, wt.%
*c*_1_	Hydrogen concentration at equilibrium, wt.%
*c_H_*	Overall hydrogen concentration in a sample, wt.%
*D*	Diffusion coefficient
*D_app_*	Apparent diffusion coefficient
*E_a_*	Activation energy
*k_0_*	Pre-exponential factor
*L*	Length of diffusion/thickness of measured sheets
*t*	Time
*x*	Position vector

**Table 4:** Pre-exponential factors and activation energies for hydrogen diffusion in pre-deformed X3CrMnNiMoN17-8-4 determined by DS cell and calculated apparent diffusion coefficients at RT according to [40].

True Strain	First Charging			Discharging		
	*k*_0_/cm^2^ s^−1^	*E_a_*/kJ mol^−1^	*D_app_* at RT/cm^2^ s^−1^	*k*_0_/cm^2^ s^−1^	*E_a_*/kJ mol^−1^	*D_app_* at RT/cm^2^ s^−1^
0	2.6 × 10^−5^	42.8	6.2 × 10^−13^	1.3 × 10^−7^	23.4	8.9 × 10^−12^
0.32	5.1 × 10^−5^	39.7	4.4 × 10^−12^	2.1 × 10^−7^	21.4	3.2 × 10^−11^
0.39	1.3 × 10^−5^	33.7	1.3 × 10^−11^	4.3 × 10^−7^	20.8	8.4 × 10^−11^
0.49	3.8 × 10^−7^	20.6	4.2 × 10^−11^	5.8 × 10^−7^	21.1	1.0 × 10^−10^

**Table 5:** Hot extraction-determined apparent diffusion coefficients for the effusion of hydrogen out of pre-deformed X3CrMnNiMoN17-8-4.

True Strain	*D_app, Hot extraction_* at RT/cm^2^ s^−1^
0	2.7 × 10^−13^
0.32	3.0 × 10^−12^
0.39	3.8 × 10^−12^
0.49	1.6 × 10^−11^

**Table 6:** Comparison of calculated and experimentally determined hydrogen amounts in different pre-strained X3CrMnNiMoN17-8-4.

True Strain	2 Weeks of Charging		2 Weeks of Discharging (Tensile Tests)	
	*c_H,exp_*/ppm	*c_H,calc_*/ppm	*c_H,exp_*/ppm	*c_H,calc_*/ppm
0	63.8 ± 17.3	64.3	35.5 ± 9.5	39.5
0.32	195.4 ± 25.0	191.7	100.6 ± 16.2	109.9
0.39	262.6 ± 35.3	271.7	190.1 ± 18.1	186.0
0.49	403.8 ± 41.1	357.5	177.2 ± 14.3	198.7

## Data Availability

The datasets presented in this article are not readily available because the data are part of an ongoing study. Requests to access the datasets should be directed to mandel@iwt.tu-freiberg.de.
